# Pamphlet Notices

**Published:** 1870-11

**Authors:** 


### Pamphlets.

*On the Origin of Diabetes; with some new Experiments regarding the Glycogenic Function of the Liver.* By W. T. Lusk M.D., Prof. of Physiology, L. I. Medical College. New York: D. Appleton & Co. 1870.

The author, after an analysis of the experiments of Bernard, Pavy, A. Flint, Jr., and a carefully devised and executed series of original experiments, concludes that the liver is the most active agent in sugar-production, but that the source of sugar is probably not confined to a single organ. Diabetes may arise from the failure of the liver to arrest the saccharine principles elsewhere generated, but passing through it, as Bouchardat has suggested. A valuable monograph.

*Proceedings of the Second Anniversary of the Nebraska State Medical Society.* June, 1870.

Two or three cases detailed to the Society may interest our readers, as suggestive of frontier professional duties.

Case 5. Reported by R. C. Moore.—William Thompson, an employee of the Union Pacific Railroad Company, was scalped by the Cheyennes, near Plum Creek Station, Nebraska, on the night of the 6th of August, 1867. He was placed under my care on the morning of the 8th, about thirty-six hours after the wounds were inflicted.

The scalp was entirely removed from a space measuring nine inches antero-posteriorly, and seven inches laterally, the denuded surface extending from one inch above the left eyebrow, backward nearly to the occipital protuberance, and laterally from one temporal region, over the vertex, to the opposite. The pericranium was in places detached, but the greater portion of that membrane was dried, and adherent to the bone. There was also a severe tomahawk wound of the right parietal bone, the fissure extending backward and downward, in the diploe, to the depth of an inch and a half, splintering the external table, but producing no injury to the internal. I also found a slight gunshot wound through the fleshy part of the right arm. The only dressing used during the whole course of treatment was surgeons' lint, saturated with pure olive oil, which excluded the air, and was easily removed for the purpose of cleaning the wound.

Healthy granulations soon appeared on the tissue surrounding the denuded calvarium, but showed no disposition to extend over the bone. In about three weeks the outer table began to exfoliate; at first at the margins, then under the adherent pericranium, the exfoliation extending more rapidly along the course of the nutrient vessels ramifying through that membrane.

As this suppurating and exfoliating process progressed, granulations sprung from the diploe, till the entire surface presented the appearance of a healthy wound. The last portions of the outer table to become detached, were the spots from which the pericranium had been removed.

The suppuration was very profuse, but the patient being strong and enjoying excellent health at the time the wounds were inflicted, did not at any time during the course of treatment present those symptoms of depression which would naturally be expected to follow so extensive an injury, nor were there any symptoms indicating that the inflammation had extended to the brain or its membranes. The only inconvenience or unfavorable complication was a severe neuralgic pain, extending down the right side of the head and face, but after the external table of the skull was cast off, the pain ceased, and there was no further disturbance of that character. The case progressed favorably, and in about three months from the time the scalp was removed, nearly the entire surface was cicatrized.

Case 7. Reported by J. H. Peabody.—G. O., Co. D., 2nd Neb. Cavalry, who was wounded by the Sioux Indians, the 22nd of June, 1863.

Mr. O. called at my office in August, 1866; his health was much impaired in consequence of the irritation caused by an arrow head which he believed was lodged in the upper portion of his right lung; when he coughed it caused great pain, and he swallowed with difficulty.

He was emaciated, and said " his life had become a burden from the constant pain and irritation kept up by the arrow head."

I found the arrow had entered to the outer side and lower margin of the right scapula, passing upwards and inwards through the apex of the lung or trachea, judging from the amount of hæmorrhage reported coming from the mouth at date of injury. Upon probing through a small fistulous opening above the superior extremity of the sternum, I found, what I afterwards ascertained to be the point of the arrow apparently firmly fixed about an inch and a half below the superior end of the sternum, lying flat against the trachea and æsophagus, with carotid artery, jugular vein and pneumogastric nerve overlaying it. With some little fear of cutting the carotid or innominata (having to tie the latter), or, perhaps worse, having my patient bleed to death in my office, I proceeded to enlarge the opening and introduced my forceps, gently lifting the arrow head from its resting place, the heel of it scraping over the arch of the aorta as it came out. Upon measurement it was found to be four and a quarter inches long, with a base three-fourths of an inch wide; rather an ugly missile to lie so long among the most delicate nerves, arteries and organs of a man's body. Mr. O. speedily regained his health, and lost his chance of a pension along with the arrow head.

Case 8. Reported by S. D. Mercer.—Result of a gun shot wound fracturing the crest of the right ilium and forming an artificial anus by ulceration of colon.

Joseph B., aged 24, strong and healthy, always lived a frontier life, and was free from that predisposition to tubercle so common among his fellow "half breeds." May 31st, 1868, he received a gunshot wound from an antagonist about ten paces in front, and a little to his right; ball entered about one inch behind the anterior superior spinous process of the right illium, fracturing crest, and probably lodged about the lower part of ascending colon. Patient reached Omaha July 3d, almost exhausted from a journey of eight hundred miles, by cars and stage, and the constant drain upon the system.

Prior to his arrival he had no treatment save the charms common among his tribe, but his father, an intelligent old Frenchman, was with him all the time, from whom the following facts were elicited. After the accident there was no movement of the bowels for ten days, when a small, hard, fæcal mass passed, containing a spicula of bone. About the same time the external wound, which had previously closed, broke, and a large quantity of pus and fæcal matter passed through this new opening. After this, the bowels only moved occasionally, but the discharge from the wound was constant. On first examination the fractured parts of the bone were found loose, and near the external opening, but it was not deemed prudent to remove them until July 14th, when, by the assistance of Drs. McClelland and Baumer, chloroform was administered, the opening enlarged, and the loose bone removed; this gave room to a large and free exit of fæcal matter and a more thorough examination of the part; injections of water per rectum were partly returned, and partly passed out through the artificial anus.

By the use of a speculum and a strong sunlight, the mucous membrane of the colon could be seen, and at one time the ileo-cæcal valves were brought plainly to sight. The wound gradually healed by granulation, the discharge grew smaller, and the general health of the patient rapidly improved; the only unfavorable symptom was retention of urine, but this was overcome by use of the catheter. Patient left Omaha for the new reservation, near Fort Randall, Aug 14th, just one month after the operation.

I am informed that he is still improving, but there is a slight discharge from the artificial anus; the right leg, rendered nearly useless by fracture of the bone to which the muscles are attached, has partially regained strength and power of motion.

### *Transactions of the Indiana State Medical Society.* 1870.

The present volume of Transactions contains several papers of intrinsic excellence. Geo. Sutton, M.D., of Aurora, Ind., the President, contributed an able and eloquent address on "Man's Power over Nature, and Medicines as means by which he aids and controls the Laws of Life."

Prof. Geo. W. Mears read a paper on the Treatment of Puerperal Hæmorrhage, which gave rise to considerable discussion. Dr. Mears: in *post partum* flooding, friction of the abdomen, grasping the fundus, and such pressure as will secure a response from within. If these do not succeed, ice water, or the cold douche to the abdomen. Or ice water intra-uterine injections, or introduction of a lump of ice, with or without a muslin cover. Or a lump of alum rubbed over the surface from which the placenta has been detached. Other astringent and styptic injections may be used, but he prefers the Persulphate of Iron, in the proportion of one ounce to eight ounces of distilled water, having first removed clots, debris, etc. Prof. Mears considers ergot indicated in these cases, but too slow in its action when given by the stomach, and suggests its use hypodermically, as recommended by Dr. Lente (he of calomel-in-sedative-doses fame.) Pressure over the descending aorta is advised in perilous cases.

Dr. G. V. Woollen, in the discussion which followed, detailed a case in which he gave Ergotine in aqueous solution, five grains in ten drops of water, by hypodermic injection inside of the thigh, with the result of very free contraction in seven minutes. He thinks it would act more promptly if more freely diluted.

Dr. Hibberd, of Richmond, has little faith in the Ergot—much in Opium.

Dr. Harvey, of Plainfield, confided, in an urgent case, in a grain of Opium, three grains of Sugar of Lead, and half a grain of Sulphate of Zinc. Considers stimulants essential—whisky in large quantities, together with aromatic Spirits of Ammonia. Thinks Ergot dangerous used hypodermically, from its forming a clot.

Dr. Rooker, of Castleton, advocated kneading the uterine region until the ball is felt, and then applying a tight bandage.

Dr. Wishard strongly encouraged the use of Opium.

Dr. Pennington, of Milton, believes that Quinine produces contraction of the uterine fibres, and expedites labor. He commences by giving from three to five or seven grains every three hours, and has " never missed getting up good contracting powers of the uterus." It is ahead of any other remedy he has ever used. In a threatening case gave whisky and five grains of quinine three times within two or three hours, with excellent results.

In the hæmorrhage of threatened abortion, Dr. Wishard uses the tampon, and Dr. Mears uses a sponge saturated with Tincture of Iron, as a direct application to the bleeding part.

E. Mendenhall, M.D., of Zionsville, follows with an essay on " The Utility of Ergot in Facilitating Labor," wherein he details the evidences, and urges the use of this very-often-doubted agent.

F. J. Van Vorhis, M.D., of Stockwell, in an essay: " Psychical Influence upon the Organization of Structures," enforces the importance of attention to mental influences in symptomatology, etiology and therapeutics. It is a philosophical glance at a subject which has been left too long to charlatans and sciolists.

H. V. Passage, M.D., of Peru, gives a history of a case of Reduction of Dislocated Hip, by Chloroform and manipulation, and R. E. Haughton, M.D., of Richmond, furnishes an essay on the Principles of the Flexion Method in the same difficulty. These papers we shall notice more in detail hereafter.

An elaborate and excellent monograph on the Pathology and Treatment of Syphilis, is given by the Secretary, G. Woollen, M.D., of Indianapolis, which deserves wider circulation than the volume of "Transactions" will secure. We would reproduce it *in extenso* did space permit. Dr. Woollen is a clear, concise and sensible writer, whose duty it evidently is to let his light shine through the medical journals.

Wilson Hobbs, M.D., of Carthage, chronicles, with illustrative plates, an interesting case of disease of the skull, requiring four operations for its successful removal—adding at least four years to the life of the patient. A remarkably large proportion of the calvarium was removed for what was apparently syphilitic degeneration of the bone.

A paper by C. E. Wright, M.D., of Indianapolis, on Purulent Aural Catarrh, a "Report on Board of Public Charities," and the inevitable report on "Medical Rank in the U. S. Navy," complete the list of published communications.

There seems to have been a very harmonious and profitable session, with a gratifying absence of discussion on points of order, the code, etc.

We congratulate our Indiana brethren on their achievement of a scientific organization and not a mere trades-union.

*Reduction of Dislocations of the Hip. Principles of the Flexion Method.* By R. E. HAUGHTON, M.D.

This is a paper published in the transactions of the Indiana State Medical Society, and is, in its scope, mainly a review of Prof. Bigelow's work on the Hip.

Dr. Haughton shows that the manipulation method of reduction was clearly recognized by Hippocrates; that it was advocated during the last century; and that flexion and rotation were intelligently combined by Despus, in 1835.

In regard to the influence of the ligamentous tissue on displacement in dislocations of the hip, and its affording an obstacle to efforts at reduction, Dr. Haughton says: "Prof. Bigelow has been anticipated." So far as the particular tissue of the iliofemoral ligament is concerned, Dr. H. quotes from Dr. Fenner's report of an autopsy in a case where hip dislocation existed, made in 1848, when that structure was "found tensely stretched," although Dr. Fenner failed to recognize the resistance which it exerted in efforts at reduction.

But Prof. Gunn's experiments and dissections, are quoted effectively to show that he had anticipated Prof. Bigelow in the influence exerted by the untorn portion of the ligament, as well as the fact that a portion of the ligament remained untorn. He quotes, also, from Prof. Moon, who repeated Prof. Gunn's experiments.

He also quotes Prof. Bigelow against himself when he admits that the "ilio-femoral ligament will *not* be found stripped clear of the remaining portion of the capsule." He further says: "the resistance is found in the ligamentous structures, mainly the capsular, which, for all purposes, includes the ilio-femoral."

But Dr. Haughton also recognizes a muscular force which he thinks aids the surgeon in his efforts to reduce by manipulation. This idea is expressed in the sixth and seventh conclusions to which he arrives, which are as follows:

"6th. That both the internal and external rotators of the hip joint are so arranged by origin and insertion, when properly directed in the flexion method, to bring the head of the bone back to this axis of motion, which passes through the acetabulum."

"7th. That the planes of the muscles, and through which they must act, are planes in all instances either parallel to the axis of motion of the hip and body, or at right angles to it."

*Transactions of the State Medical Society of Pennsylvania. Twenty-first Annual Session.* 1870. Sixth Series, Part I, Pp. 216.

From the address of welcome by Dr. J. A. Meigs, of Philadelphia, we cannot forbear to extract the following judicious remarks:

"It must never be forgotten that medical organizations in this country, and especially in this State, have no legal, but simply a moral power. The due appreciation of this fact is more than ever necessary now, since it is evident that in the medical profession the strongest obstacle to its thorough and efficient organization is the tendency to individualization which is manifestly growing stronger every day. This tendency, so clearly attributable to the large increase in individual liberty resulting from the career of development through which civilization has for centuries been slowly advancing, is heightened by the too exclusive cultivation of specialties in medical science, and by the low standard in medical education, and the consequent introduction into the ranks of medicine of incompetent persons, who, conscious of their lack of skill, and pressed by the intense competition for business, resort to practices to insure pecuniary success which are completely subversive of all dignity in medicine as a liberal profession.

"It being apparently impossible to overcome the difficulties just indicated by mere legislative action on your part, it may well be asked whether much of the time spent in the discussion of the ethical relations of the profession would not be far better devoted to other, and, perhaps, after all, the greatest objects of your Society—objects which, in the second article of your constitution, are defined to be "the extension of the bounds of medical science and the promotion of all measures adapted to the relief of suffering, the improvement of the health, and the protection of the lives of the community." Under present circumstances, the elevation of professional character and the protection of your individual interests can only be accomplished by the practical recognition of the scientific requirements of medicine. So long as medicine continues to be purely empirical, so long will quackery continue to flourish. Not by exposing its criminal falsifications can you make quackery impossible, but rather by casting the strong light of science upon the doubtful and obscure places in medicine. Only under the influence of this light can the mercenary impostors who cajole the public, and make the practice of physic a mockery and a by word, be driven away as the mists of night before the coming dawn. In the cultivation of medical science lies your true and only strength. By cultivating science, you give to medicine exactitude; by giving to the healing art exactness, you place it upon an elevated platform; you isolate at once this goodly tree from the horde of pretenders who so long have carried on their nefarious schemes under the benign influence of its widely-spreading branches. You all know how surgery, resting upon the exact science of anatomy, and calling to its aid various accurate mechanical appliances, is now but little troubled with impostors. The "natural bone-setters" and other similar charlatans have long since disappeared from its domain. You also know how practical medicine, on the other hand, based as it is upon an experience which is so often fallacious, upon a physiology and pathology still very imperfect, and an organic chemistry in a state of great confusion, is still a prey to the rapacious cunning of every quack who boasts his infallible remedy for phthisis, rheumatism, and all those special ills which, in consequence of our ignorance of their essential nature, continue to resist all therapeutic effort. The problems of medical science awaiting solution are numerous.

The difficulty of their solution is equaled only by its importance. No more important object, indeed, can occupy your thoughts and time than the systematic attempt to clear up some at least of these questions; many of them can be investigated by the clinical methods peculiar to medicine; for the unraveling of others, aid must be sought in the methods and appliances of physical, chemical and natural science. There is ample room for the gratification of every taste, of every mental tendency. Look around you and behold with what extraordinary rapidity science is unfolding itself in a multiplicity of directions. To practical medicine the momentum of this active development has been imparted with happy results. By means of various ingenious scientific instruments, such as stethoscopes, laryngoscopes, ophthalmoscopes, otoscopes, endoscopes, microscopes, thermometers, manometers, the sphygmograph of Marey, the stetho-sphygmograph of Hawkins, different electrical machines, etc., the laws of sound, light, heat, electricity and mechanics have been practically employed, in not a few instances, with signal success, in elucidating the phenomena of disease. In consequence of this rapid advance in science, and the multiplication of scientific instruments, medicine is at present undergoing a remarkable change. While its data are daily becoming more and more exact, the theories or fundamental principles which constitute its framework, so to speak, are undergoing, like our social fabric, a complete revolution. To the reflecting mind it is evident that medicine is now passing through a chaotic phase in its onward career. The day of blind obedience to authority is at an end. No asserted fact, no theory however plausible, finds its way to acceptance on account of the great name attached to it, but, on the contrary, is immediately tried in the crucibles of experiment, observation and induction, by that earnest and enthusiastic band of laborers, who, whether in physics or biology, are seeking with busy hands to reconstruct the philosophy of medicine and place it upon a sure, scientific basis."

In another department of the present number will be found several practical scissorings from this interesting volume.

*Transactions of the Medical Society of New Jersey*, 1870. Pp. 214.

From this pamphlet we find room only for the following extracts. Dr. B. Rush Bateman, of Cumberland Co., reports:

"In one family we were present at five successive births, where each child was a hermaphrodite, partly composed of both sexes, especially the genital organs. In every case there was a penis and vagina, in two of which there was a sack on each side of the vagina externally, with a testicle in each. It was exceedingly difficult to classify the sex in such cases. In one case in particular, were both sexes so well defined, that it was finally concluded to class it with the males; it was called Charles. As it grew older it was thought it would, by its instincts, develop its sex, by wanting a knife, a doll or needle, and so it did; for when a proper age arrived to choose its articles of amusement, it wanted a doll, needle and thread; its name was then changed to Charlotte. She is still living and healthy, thirty or more years old. Her features are coarse, voice more masculine than otherwise; but is smart and active, said to be remarkable for her sagacity at housewifery; does not mix with either sex to any extent.

"All of the five were healthy children, and lived to grow to maturity; in fact, all are now living but two. One was married, but he never had any issue."

Dr. Charles F. J. Lehlbach gives clinical observations on Scarlatina, with remarks. This is illustrative:

"CASE IV.—Passing a neighborhood where I had been attending several cases of Scarlet Fever, I was called in to see a girl, aged 12 years, who had, so I was told, "caught a cold." Found her with some soreness of throat, fever, and a slight scarlet rash. Her little brother was then convalescent from a very mild attack of the disease. Pronounced the case Scarlet Fever. Prescribed, and ordered the child to remain in the house. Heard no more of the patient until, in the afternoon of May 27th, 1869—two weeks after I first saw her—I was called in great haste, as the child had spasms, and "would probably be dead before I could get there." Found her in a really alarming condition. Violent spasmodic contractions of upper and lower extremities, alternating with rigidity of the muscles, partial opisthotonos, foaming at the mouth, teeth tightly set, eyes vacantly staring, entirely unconscious, pupils somewhat dilated, reacting very feebly, breathing rapid and laborious, skin preternaturally hot.

"By a few rapid questions I learned that my injunctions to stay at home had only been followed for a few days. She had then resumed her attendance at school, although not feeling entirely well, until some days previously, when on returning from school, nearly a mile distant, she had been caught in a heavy storm and been thoroughly drenched. With the exception of a little fullness about the face, no anasarca. Urine had been scanty.

"*Diagnosis.*—Toxæmia, imperfect elimination of Scarlet Fever poison, probability of renal trouble, congestion and transient œdema of the brain, with serous apoplexy imminent.

"*Indications.*—Revulsives, nervous sedatives, reëstablish the function of the kidneys. Sent for mustard, ice, chloroform, and bromide of potassium.

" *Treatment.*—Mustard to feet and legs; crushed ice, wrapped in a towel, under the spine, ice to the head; rub chest and abdomen with ice; by mouth 30 drops of chloroform with a scruple of bromide of potassium, followed in half an hour by one drachm of chloroform, and one drachm of the bromide, suspended in milk, per anum. Then suspended the ice applications, wrapping the patient in woolen blankets. In the course of an hour and a half the spasms recurred at longer intervals, with less intensity; muscles relaxed; in two hours she was able to swallow well, and showed signs of returning consciousness. An hour later, perfectly conscious, with no sign of cerebral disturbance except total blindness, of which she complained. Left her, with directions to renew sinapisms and to take ten grains of bromide of potassium every three hours until natural sleep ensued, and to be called if there was a change for the worse. The next day I found her perfectly rational—exhausted—no fever—pulse a little frequent—blindness had continued nearly ten hours when sight reappeared—urine slightly albuminous. Ordered lime water and digitalis, quinia, and tr. ferri. Four days later, urine free, no trace of albumen. Continue tonics. Patient discharged."

Dr. T. F. Morris illustrates Puerperal Eclampsia by cases of which this is one:

" Mrs. P.—April 28, 1869, called to see Mrs. P., pregnant for the second time, expecting her confinement, at the latest, during the early part of May. Rather full habit; was complaining of cephalalgia, obscurity of vision, and general lassitude; extremities slightly œdematous; face somewhat puffy. She had taken a saline cathartic before sending for me; ordered it repeated. Requested her to keep for me the morning following, her urine, for examination. During the night she was seized with a convulsion, and being in attendance upon another case, my friend, Dr. Reeve, saw her. Had her cupped on temples and back of neck, which was being done when I arrived. Upon digital examination the os was found to be dilating and uterine effort quite regular. These spasms continued during the whole night and through the following morning, with no return of consciousness. Chloroform was used cautiously and carefully, to suspend these terrible convulsions, and did seem to moderate their violence very much. Elaterium was given, which produced free catharsis. Bromid. potass. was given by injection, ʒj. doses, every hour. Diaphoresis produced by wrapping hot bricks with wet cloths, and placing them about her person. Her labor terminated about 2 P. M., of the 29th. All convulsive movements ceased. She remained comatose during the following night, and with the morning hours reason again returned. She was still kept upon the bromide, ʒss. every three hours, until the following day, when considerable dyspnœa manifesting itself, her lungs were examined, and pneumonia was found, to complicate her troubles. She was then put upon 10 grs. Iodide potass. every 2 hours for the next 48; her chest enveloped in an oiled silk jacket, and at the end of this time the dose of the Iodide was lessened to 10 grs. every 3 hours; and finally, after two days more had elapsed, Iodide was reduced to 5 grs. every 4 hours. She was freely stimulated, fed with ess. Bf., and after a hard struggle for life, convalesced. I omitted to state that her urine was largely albuminous, and continued to manifest this condition until convalesence was positively established."

